# Decreasing delirium through music listening (DDM) in critically ill, mechanically ventilated older adults in the intensive care unit: a two-arm, parallel-group, randomized clinical trial

**DOI:** 10.1186/s13063-022-06448-w

**Published:** 2022-07-19

**Authors:** Sarah Seyffert, Salwa Moiz, Matthew Coghlan, Patil Balozian, Jason Nasser, Emilio Abi Rached, Yasser Jamil, Kiran Naqvi, Lori Rawlings, Anthony J. Perkins, Sujuan Gao, J. Downs Hunter, Sikandar Khan, Annie Heiderscheit, Linda L. Chlan, Babar Khan

**Affiliations:** 1 Department of Medicine, IU School of Medicine, Indianapolis, IN USA; 2 IU Center of Aging Research, Regenstrief Institute, Indianapolis, IN USA; 3 Lebanese American University, Beirut, Lebanon; 4 Department of Biostatistics and Health Data Science, Indiana University School of Medicine, Indianapolis, IN USA; 5 Area 10 Labs LLC, Rochester, MN USA; 6 Division of Pulmonary, Critical Care, Sleep and Occupational Medicine, Department of Medicine, Indiana University School of Medicine, Indianapolis, IN USA; 7 Department of Music, Augsburg University, Minneapolis, MN USA; 8 Department of Nursing, Nursing Research Division, Mayo Clinic, Rochester, MN USA

**Keywords:** Delirium, Music, Post-intensive care syndrome, Acute respiratory failure, Mechanical ventilation, Cognition, Intensive care unit, Pain, Anxiety

## Abstract

**Background:**

Delirium is a highly prevalent and morbid syndrome in mechanically ventilated intensive care unit (ICU) patients. Music is a promising non-pharmacological intervention with beneficial effects on anxiety and stress, while its effects on delirium duration and severity are not well understood.

**Methods/design:**

Our study is a two-arm, randomized parallel-group, clinical trial to evaluate the efficacy of music intervention compared to a silence-track attention control on delirium/coma duration in mechanically ventilated critically ill older adults.

One hundred sixty mechanically ventilated adults 50 years of age or older will be randomized to one of two arms within 72 h of ICU admission: (1) 1-h music listening sessions twice daily through noise-canceling headphones, or (2) 1-h sessions of a silence track twice daily through noise-canceling headphones. Our primary aim is to compare delirium/coma-free days after randomization during the 7-day study intervention phase using the Confusion Assessment Method for the ICU (CAM ICU) and the Richmond Agitation Sedation Scale (RASS) for delirium and coma. Secondary outcomes include pain and anxiety evaluated twice daily during the intervention phase and throughout the duration of ICU stay using the Critical Care Pain Observation Tool (CPOT) and visual analog scale-anxiety (VAS-A). Enrolled participants will be followed after hospital discharge to further measure cognition as well as screening for depression and anxiety using the following telephone-based instruments: Indiana University Telephone-Based Assessment of Neuropsychological Status (IU TBANS), Patient Health Questionnaire-9 (PHQ-9), and Generalized Anxiety Disorder-7 (GAD-7).

**Discussion:**

This randomized clinical trial will measure the efficacy of a music listening intervention for delirium and coma duration early in the intensive care unit among older adults.

**Trial registration.:**

ClinicalTrials.gov. NCT04182334.

**Supplementary Information:**

The online version contains supplementary material available at 10.1186/s13063-022-06448-w.

## Background

Delirium is a syndrome of acute brain dysfunction characterized by a disturbance of consciousness, the presence of inattention, disorganized thinking, and a fluctuating course [1]. One million adults in the United States receive mechanical ventilation for acute respiratory failure in intensive care units (ICUs) annually, and up to 80% of them develop delirium during their stay. Delirium predisposes older adults to immediate in-hospital complications including longer length of ICU and hospital stay, increased risk of inpatient mortality, and elevated costs of care [2–6]. It is also associated with long-term post-ICU complications including cognitive impairment and dementia [7]. Mechanically ventilated patients experience pain and anxiety which is often managed by the administration of sedative agents that are independently associated with the development of delirium [8–15]. Despite its prevalence, effective pharmacological interventions for delirium are not available. Previous use of pharmacological agents including haloperidol, ziprasidone, and acetylcholine lowering medications have not been shown to be superior to placebo [15] further corroborating the need for nonpharmacological therapies that may prevent or manage delirium.

Prior music listening research by Chlan et. al has demonstrated a reduction in anxiety, pain, sympathetic nervous system activity, and the attenuation of inflammatory mediators in critically ill mechanically ventilated patients [16]. While this provides a theoretical framework that neuroinflammation could be reduced by music, it has not yet been established that music reduces delirium in ICU patients who are mechanically ventilated.

We recently conducted a three-arm pilot acceptability and feasibility trial among mechanically ventilated, critically ill patients (MPIs: Khan and Chlan) testing two music listening interventions (patient-preferred or slow-tempo relaxing) delivered through iPads and noise cancelation headphones against an audiobook attention control [17]. The trial not only showed high feasibility and acceptability of 80% in each music arm, but also demonstrated a signal towards reducing delirium duration in patients assigned to the slow-tempo relaxing music group. This current protocol for a follow-up randomized clinical trial (DDM: *Decreasing delirium through music in critically ill, mechanically ventilated older adults*) tests the efficacy of a 7-day music listening intervention in reducing delirium and improving post-ICU brain health among critically ill, mechanically ventilated older adults.

## Methods/design

### Design

DDM is a two-arm, randomized parallel-group efficacy clinical trial to test superiority of music listening intervention compared to a silence track attention control in reducing delirium duration and severity in mechanically ventilated critically ill older adults. The total duration of the intervention will be 7 days from randomization unless a patient has died, discharged, or transferred out of the ICU.

The randomized clinical trial is written according to the Standard Protocol Items: Recommendations for Interventional Trials Statement (SPIRIT). A SPIRIT Table is provided and a SPIRIT Checklist is provided (see Table 1 and Additional file 1).

**Table 1:** Standard Protocol Items: Recommendations for Interventional Trials (SPIRIT) figure for the DDM trial

Timepoint	Study period						
	Enrollment	Allocation	Post-allocation				Close-out
	− t	0	Day 1–7	Day 8–14	Day 15–21	Day 21–28	Day 90
Enrollment:							
Eligibility screen	x						
Informed consent	x						
Demographics, comorbidities, functional status, clinical data, baseline cognitive function (IQCODE), diagnoses, severity of illness, functional status, medications	x						
Allocation		x					
Interventions:							
Slow tempo music		2x	2x				
Attention control		2x	2x				
Assessments:							
ICU assessments: RASS, CAM-ICU, CAM-ICU7, CPOT, VAS-A	x	x	2x	2x	2x	2x	
IUTBANS, PHQ-9, GAD-7							x
Other assessments:							
Clinical data	x		x				
Discharge disposition			x	x	x	x	
Intervention fidelity			x				
Physiologic parameters	x		4x	x	x	x	
Length of stay and healthcare utilization							x

Abbreviations: *CAM-ICU* Confusion Assessment Method for the Intensive Care Unit, *CPOT* Critical Pain Observation Tool, *RASS* Richmond Agitation-Sedation Scale, *VAS-A* visual analog scale-anxiety, *IUTBANS* Indiana University Telephone-Based Assessment of Neuropsychological Status, *PHQ-9* Patient Health Questionnaire, *GAD-7* Generalized Anxiety Disorder Scale

### Setting

Patients are screened at multiple hospitals associated with Indiana University School of Medicine, including Sidney and Lois Eskenazi Hospital, a 618-bed urban public hospital, and Indiana University Health Methodist Hospital, an 802-bed quaternary care hospital. Patients will be recruited from mixed medical and surgical ICU units (65 beds) to increase the generalizability of our findings.

### Participants

The target population for our trial is adults aged 50 years and older admitted to the medical or surgical intensive care unit and expected to be on mechanical ventilator support for 48 h or more.

### Inclusion criteria

Inclusion criteria of our study are as follows: (1) age 50 years or older, (2) English speaking, (3) admitted to the intensive care unit (medical or surgical), (4) expected to be on mechanical ventilator support for at least 48 h, (5) able to provide consent through a legally authorized representative, and (6) have access to a telephone after hospital discharge.

### Exclusion criteria

Patients are excluded from our study if any of the following conditions are present: (1) history of dementing illnesses and other neurodegenerative diseases such as Alzheimer’s disease or vascular dementia; (2) severe psychiatric illness which is not well controlled; (3) alcohol withdrawal symptoms or concern for withdrawal symptoms; (4) suspected or confirmed drug intoxication/overdose; (5) acute neurologic injury such as traumatic brain injury, ischemic or hemorrhagic cerebrovascular accident, or undergoing neurosurgery; (6) uncorrected hearing or vision impairment including legal blindness; (7) incarcerated at the time of study enrollment; (8) enrolled in another clinical trial which does not permit co-enrollment; or (9) any medical condition precluding safe use of headphones such as skin breakdown, burns, facial, or skull fractures.

### Consent

After reviewing electronic medical records, research staff will approach eligible patients or legally authorized representatives for consent. If on mechanical ventilation and unable to consent, the participant’s legally authorized representative will consent. The patient will be re-consented once clinically able.

### Study procedures

#### Screening

Patients admitted to the ICU will be screened for eligibility by trained research personnel and the patient or legally authorized representative(s) approached for informed consent if meeting eligibility criteria.

#### Participant randomization

Participants will be randomized using a computer-generated randomization list created by a study statistician by permuted block with varying block sizes. Randomization to one of the two study arms will occur after consent in a 1:1 manner and will be stratified based on hospital location. Randomization assignment will be performed by an unblinded study coordinator who does not perform any intervention or outcome assessments.

#### Intervention duration/termination

**Fig. 1:**
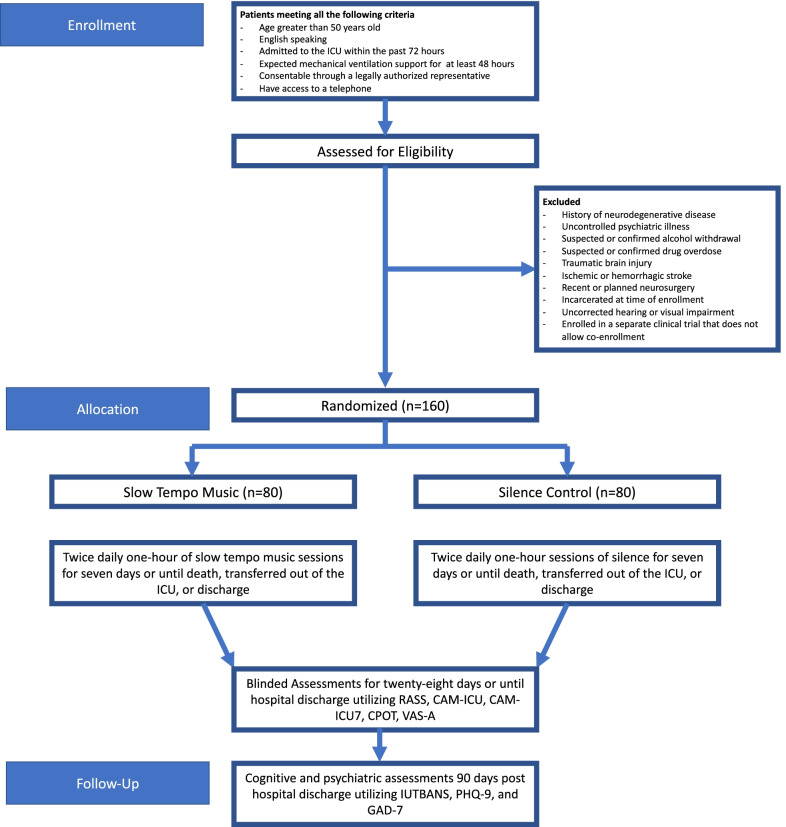
DDM study flow chart, interventions, and assessments. Abbreviations: CAM-ICU, Confusion Assessment Method for the Intensive Care Unit; CPOT, Critical Pain Observation Tool; RASS, Richmond Agitation-Sedation Scale; VAS-A, visual analog scale-anxiety; IUTBANS, Indiana University Telephone-Based Assessment of Neuropsychological Status; PHQ-9, Patient Health Questionnaire; GAD-7, Generalized Anxiety Disorder Scale

The intervention period will be up to 7 days post randomization. Participants will stop receiving intervention if they withdraw, transfer out of the ICU, are discharged, or die. In-hospital follow-up continues for 28 days or until discharge, whichever occurs earlier, by daily assessments as shown in Fig. 1.

### Materials

Experimental and attention control conditions will be delivered through identical iPads with music tracking app and noise cancelation headphones to ensure blinding when study staff perform assessments. A custom application (app) platform will be used that has been developed by Area 10 Labs, a biomedical development company located in Rochester, Minnesota, that has been pilot tested for feasibility by our group. The application will be downloaded on study iPads and will automatically capture highly granular data about daily listening frequency, duration, and music selections. Also, an extensive library of slow-tempo 60 to 80 beats/minute music selections developed under the expert guidance of a board-certified music therapist (Dr. Heiderscheit) will be delivered. The playlists will be loaded onto dedicated study iPads to offer a wide variety of music choices and individual preferences. Utilizing automatic electronic data capture on iPads will result in a low resource, ready to use intervention that can be integrated into routine patient care in any ICU.

### ICU intervention phase

#### Experimental condition

Music listening sessions will take place twice a day, for 1 h at a time between 07:00 and 12:00 and between 13:00 and 20:00 for up to 7 days. The times are based on feedback from ICU nurses associated with the least interference of daily clinical activities. Music listening will be provided for 7 days unless the participant refuses, withdraws, is transferred out of the ICU, or dies. Participants extubated within the 7-day period will continue to receive the intervention unless they are transferred out of the ICU. Participants remaining in the ICU after 7 days will continue to undergo further assessments of delirium until 28 days of ICU stay, but the active intervention will be discontinued after day 7. The 7-day intervention duration was carefully determined based on our prior delirium trials demonstrating that delirium occurs early during the ICU stay with mean duration of delirium of 3.4 days in the PMD trial, median delirium duration of 4 days in the MIND-USA trial, and the mean delirium duration of 3.1 days in the DDM pilot trial [15, 17, 18].

Twice daily 1-h intervention will be delivered based on our extensive playlist created by board-certified music therapist consultant (Dr.Heiderscheit). In addition to the twice daily music intervention sessions, patients will be encouraged to self-initiate listening to slow-tempo music from the study playlist whenever desired as a means to engage in quiet time or listening enjoyment. Our iPad app will efficiently log music listening sessions by automatically capturing frequency, length, and music selections for both the twice daily music intervention sessions as well as the self-directed listening sessions.

#### Control condition

Patients randomized to this arm will receive a noise cancelation headphone-applied condition twice daily in 1-h sessions identical to the music intervention arm. Likewise, patients will have the opportunity to use the silence app with headphones whenever desired. As with the music intervention, the app will track each session’s length, frequency, and self-directed headphone application with the silence track.

#### Other data collection

**Fig. 2:**
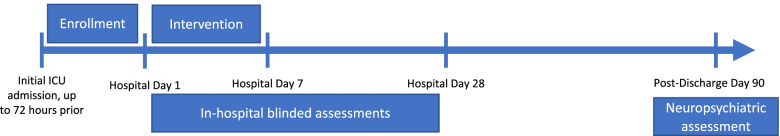
Timeline of study events

Physiological parameters of respiratory rate, heart rate, and blood pressure will be collected four times daily before and after the assigned intervention during the 7-day intervention phase and daily afterwards for the duration of the ICU stay; see Fig. 2.

As an ancillary component of this study, blood samples from participants will be collected and stored for future analyses. Samples will be obtained during the hospital phase of the study; additional details are outlined in the appendices.

#### Blinding during intervention

We will use various strategies to ensure concealment of a participant’s allocation. Only the unblinded study coordinator will program the audio devices. Participants, their family members/surrogates, and hospital providers are asked not to share a patient’s randomization with anyone in the study, should they become aware of it. Audio is not turned on until the headphones are securely placed on the participant to prevent study personnel from inadvertently hearing the output. Participants will be permitted to change tracks or adjust volume if needed. Should one of the study personnel become unblinded, they will be reassigned to other study participants.

### Outcomes

The primary outcome of improving delirium is measured by delirium/coma-free days. Delirium/coma-free days is defined as the number of days after randomization patients are alive, free of delirium, and not in coma during the 7-day study ICU intervention phase. Delirium severity is assessed by trained research personnel using the CAM-ICU-7 [3, 19]. Symptoms of pain and anxiety are also primary outcomes. Pain is assessed by the Critical Care Pain Observation Tool (CPOT) and anxiety intensity is assessed by the vertical visual analog scale-anxiety (VAS-A).

The secondary outcomes of the study are assessment of music’s effects on cognition at 3 months after hospital discharge measured by memory, attention, information processing, speed, and executive cognitive functioning using the Indiana University Telephone-Based Assessment of Neuropsychological Status (IU TBANS). An assessment of depression and anxiety using the Patient Health Questionnaire 9 (PHQ-9) and the Generalized Anxiety Disorder 7 (GAD-7) will also be collected over the telephone to determine the impact of music intervention on ICU survivors’ mood and anxiety.

#### Assessments during ICU intervention

All assessments are performed by blinded research personnel. Assessments occur twice daily between hours of 09:00 to 12:00 and 14:00 to 20:00 (after audio interventions). All enrolled patients will receive standard ICU care based on local practices. Patients will continue to receive clinical care for delirium based on their primary clinical team’s discretion. Study staff will assess for delirium, pain, and anxiety for the duration of the ICU stay or until day 28 if the patient remains in the ICU. Study staff perform the following: (1) RASS to document the level of consciousness; (2) CAM-ICU to screen for delirium, and (3) CAM-ICU7 to determine delirium severity [3, 19–21]; (4) CPOT to determine pain; and (5) VAS-A to determine self-reported anxiety intensity.

#### Assessments post ICU intervention

Participants receive in-hospital assessments by blinded research personnel until hospital discharge, day 28, withdrawal from the study, or death. Twice a day, study personnel will perform the RASS, CAM-ICU, CAM-ICU7, CPOT, and the VAS-A, while the patient remains in the ICU. Patients will be monitored for adverse events daily.

#### Safety and adverse events monitoring

Adverse events will be reported to the Institutional Review Board (IRB), data safety monitoring board (DSMB), and contact MPI, Dr. B. Khan, immediately. In our prior work, we have not experienced any adverse events related to the study protocols. All infection control guidelines will be followed based on each hospital’s policy. Patient risks will be minimized through use of quality electronic devices, regular cleaning and maintenance. Lastly, given the high-intensity nature of the ICU, all patients will receive benefit from frequent monitoring in the ICU as part of routine care. The data monitoring plan includes the appointment of an independent safety officer (SO) to perform data and safety monitoring activities. The SO will advise the PIs and the NIA program officer, regarding safety, study risks and benefits, scientific integrity, participant recruitment, and ethical conduct of the study. The SO will review the reports sent by the study manager to determine whether there is any corrective action needed, including data audit and review.

Data will be linked to participants through the use of a unique identifying number. Only persons on the research team will have access to the data. All data are collected for research purposes only. Case Report Forms (CRFs) will be stored in locked filing cabinets at Regenstrief Institute and all data will be entered into electronic report forms (eCRFs) in a secured password-protected database. All study data will be entered via a password-protected study-specific REDCap (Research Electronic Data Capture) database website. REDCap was developed specifically around HIPAA security guidelines and has been disseminated for use locally at other institutions and currently supports > 140 academic/non-profit consortium partners and 11,000 research end-users (www.project-redcap.org). Data will be retained in accordance with NIH guidelines.

#### Follow-up after hospital discharge

Post hospital discharge, assessments are obtained at 3 months. Cognition will be measured by Auditory Verbal Learning (AVLT), Digit Span, Symbol Digital Modalities Test (SDMT), and Controlled Oral Word Association test (COWA) using a telephone-based administration format, the IU TBANS. Previous research has demonstrated that these tests can be delivered reliably and precisely by telephone. The AVLT is a five-trial fifteen-item word list learning task that provides two scores: sum recall of the five learning trials and delayed recall. Digit Span measures working memory and the ability to repeat short random number sequences forward and backward. We will use the oral version of SDMT. Participants will be mailed the SDMT response sheet prior to the assessment and told to keep the envelope sealed and at hand for the day of the assessment. At the day of the assessment, the participant will be instructed to open the envelope and the SDMT practice items will be completed. Total score is the number of correct numbers-symbol pairings called out in 90 s. The COWA is a measure of verbal fluency and executive cognition function in which words are generated orally that begin with specified letters of the alphabet. Scores derived from these tests can be analyzed separately or combined to form a single composite score. We will provide participants with pre assessment instructions to structure the assessment to minimize distraction and interruption and to maximize reliability and precision.

PHQ-9 and GAD-7 will also be used. The PHQ-9 is a nine-item depression scale with a total score from 0 to 27 and the GAD-7 is a seven-item anxiety scale with a total score from 0 to 21. At the completion of the follow-up period, patients seeking additional post-ICU care can be evaluated at the Indiana University Critical Care Recovery Centers.

The study will benefit from highly trained research staff familiar with best practices to optimize recruitment and retention. Our research team comprises culturally sensitive, diverse research staff that promote enhanced communication and comfort of study participants. Retention throughout the study will be monitored in the following ways: weekly team meetings to discuss recruitment and retention challenges and strategies, close partnership between research staff and clinicians, extensive training in ethical recruitment techniques, and frequent communication between participants and study team members to build a relationship. As clinical research in the ICU can be challenging, the study team will closely monitor attrition rates, adherence to intervention, and completion rate of outcome assessments.

### Data collection

Study data will be collected and managed using Research Electronic Data Capture (REDCap®) hosted at Indiana University. REDCap® is a secure, web-based application designed to support data capture for research studies. During all phases of the study and for up to 28 days, blinded personnel collect physiological vital signs, pertinent laboratory and imaging data, data to calculate the severity of illness, clinical events including surgery and tracheostomy, duration and modes of mechanical ventilation, cumulative daily doses of sedatives, analgesics, antipsychotic medications, shock requiring vasopressors, and the administration of systemic steroids or paralytic agents. Disposition at discharge will also be collected.

### Sample size and feasibility

Sample size estimation is based on the observed effect size from our pilot data where the intervention group had higher delirium/coma-free days than the control group with estimated effect size of 0.52 [17]. Using two-sample *t*-test, we estimate that we will need 128 total patients with complete data to detect an effect size of 0.5 SD or higher at *α* = 0.05. For robustness of results, we also estimated power if we assume Poisson distribution for mean delirium/coma-free days with a total 128 patients observed at day 7, we will have 98.7% power to detect group difference at *α* = 0.05 using the nonparametric Wilcoxon test. Allowing up to 20% attrition rate for patients dropping out before day 7, we will enroll 160 total patients for this trial (80 per group).

For anxiety measured by VAS-A, we estimated power using results published by Chlan et. al where the author reported that changes in VAS-A in both the music and control groups appear linear and the noise-canceling headphones group had no-significant change over time [9]. Assuming that 128 patients complete anxiety evaluation by day 7, a daily decrease of 0.03 SD on VAS-A in the music group and no change in the control group, we will have 83.5% power in detecting a group and time interaction in the mixed effects model at *α* = 0.05 assuming a first order autoregressive correlation structure with 0.4 for measurements one day apart. For pain, assuming a baseline mean of 0.54 for both groups (SD = 0.63), a daily decrease of 0.2SD on CPOT in the music group, and no change in the attention control group, we will have 80.6% power to detect a group and time interaction in a mixed effect model assuming the same covariance matrix as for VAS-A. We will also have 80.6% power to detect a significant group by time interaction assuming similar CAM-ICU-7 trajectories to CPOT in the two groups. For cognitive scores, PHQ-9 and GAD-7, we will have 80% power to detect an effect size of 0.5 SD between the two groups using a two-sample *t*-test at *α* = 0.05. The power estimates were conducted using the Power and GLM Power procedures in SAS 9.4.

### Statistical analysis

We will compare patients’ demographic characteristics (age, gender, race, and levels of education) and illness severity between the intervention and control groups using two-sample *t*-tests for continuous variables and chi-square tests for categorical variables to determine whether the two groups are balanced in these variables. Variables that are found to be unbalanced will be included as covariates in all models comparing outcomes between the two groups. We will use an intention to treat approach in all analyses. For our primary specific aim, delirium/coma-free days by day 7 will be compared using analysis of covariance (ANCOVA) model with a group (intervention versus control) as the independent variable while adjusting for the baseline patients’ demographic and clinical variables (illness severity). For patients discharged before day 7, the days from discharge to day 7 will be counted as delirium/coma-free. For patients who died before day 7, zero delirium/coma-free days will be counted from date of death to day 7. We have used the same approach in our previous trials and this approach is able to appropriately handle potential missing data due to discharge or death of patients before day 7 [18].

Mixed effects models will be used to compare delirium severity (CAM-ICU7), pain (CPOT), and anxiety scores (VAS-A) measured twice daily from randomization to day 7, time of death, or discharge. In each of the models, repeated CAM-ICU-7, CPOT, or VAS-A scores will be the outcome variable respectively. Group, time, group, and time interactions will be included as independent variables while adjusting for potential baseline covariates. Significant group and time interactions will indicate differences in changes of these outcomes between the two groups. Post hoc analyses will be conducted to determine the earliest times when significant differences between these outcomes can be detected. We will compare various variance covariance structures including autoregressive (AR) and variance components blocked by the time of the day (am versus pm) using likelihood ratio tests to determine the most appropriate covariance structure. We will also examine potential non-linear trajectories of change in these outcomes by including quadratic time in the models and by fitting spline models. ANCOVA models will be used to compare IU-TBANS, PHQ-9, and GAD-7 collected at 3 months post hospital discharge with the group as the independent variable while adjusting for potential baseline covariates.

#### Missing data

We expect up to 20% of patients may not complete day 7 due to death in the ICU or discharge before day 7. We will conduct sensitivity analyses with varying assumptions for the patients with missing outcomes to determine the robustness of our findings. The mixed effects model we propose for repeated measures is robust under the missing at random assumption and provides an unbiased estimate of the intervention effect if we include all relevant covariates in the model. We will also compare baseline characteristics of patients with a 3-month assessment and those who are lost to follow-up and adjust for the variables associated with missing data to ensure unbiased estimation and inference.

## Discussion

DDM is a two-arm randomized clinical trial testing the efficacy of music listening intervention in improving delirium/coma-free days among critical mechanically ventilated patients as compared to attention control. Our protocol builds on strong prior research findings of music’s anxiolytic properties [10]. Music listening interventions for hospitalized and non-hospitalized patients have shown decreases in heart rate and blood pressure hypothesized to be a consequence of a lower sympathetic drive [10, 12]. Earlier studies have also shown lower sedative doses for patients undergoing procedures and utilizing preferred music listening [9]. Prior studies have been limited by methodological challenges, including small sample size, lack of blinding, and exclusion of the critically ill. The strengths of our protocol include blinding of outcome assessors, twice daily delirium assessments, as well as assessments of pain, anxiety, and cognition post hospital discharge. Furthermore, our study benefits from high external validity through inclusion of the ICU’s most critically ill. Music listening has been shown to activate areas of the brain involved with memory, cognitive function, and emotion [22, 23]. By reducing brain dysfunction and increasing activity in the areas related to memory, music could help retain cognitive function, particularly in older people who experience critical illness or injury. As such, results from our trial may permit development of music algorithms and implement music listening protocols in a busy ICU.

## Supplementary Information

**Additional file 1.**

**Additional file 2.**

## Data Availability

Data results from this study are unavailable at the time of publication. Access to the final dataset will be retained with the study investigators. Compensation for the trial, including harm, is not intended. Results of the trial are intended for publication in a peer-reviewed journal, by the authors, and assistance of professional writers is not expected. Results will be shared with participants of the study by email and within our hospital system. Full access to the protocol or participant-level dataset is available upon request.

## Abbreviations

RASS: Richmond Agitation Sedation Scale
CAM ICU: Confusion Assessment Method in the ICU
CPOT: Critical Care Pain Observation Tool
VAS-A: Visual analog scale-anxiety
IU TBANS: Indiana University Telephone Based Assessment of Neuropsychological Status
PHQ-9: Patient Health Questionnaire
GAD-7: Generalized Anxiety Disorder Scale
