# Supplementary material for: Decreasing delirium through music listening (DDM) in critically ill, mechanically ventilated older adults in the intensive care unit: a two-arm, parallel-group, randomized clinical trial
Source: Trials. 2022 Jul 19;23:576. doi: 10.1186/s13063-022-06448-w (PMC9295531; doi:10.1186/s13063-022-06448-w)
Supplement: Supplementary file 1 — Additional file 1. [file 13063_2022_6448_MOESM1_ESM.docx]

# DECREASING DELIRIUM THROUGH MUSIC

# IN CRITICALLY ILL OLDER ADULTS

# DDM

**Data Safety Monitoring Plan**

Grant Title: **Decreasing Delirium through Music (DDM) in critically ill older adults**

Grant #: ***1R01AG067631-01***

Study Title: **DDM (Decreasing Delirium through Music)**

IRB #: [1906664366](https://apps.iu.edu/kc-prd/kr/inquiry.do?protocolId=31745549&businessObjectClassName=org.kuali.kra.irb.Protocol&methodToCall=start)

**Table of Contents**

1. **Introduction**
2. **Organization**

# Qualification and Responsibilities of the Safety Officer

1. **Responsibilities of the DDM Study Team**
2. **Conduct of Data Safety Review meetings**

# Expedited Safety Reporting

# Protection of Human Subjects

# Introduction

One million adults in the United States receive mechanical ventilation for acute respiratory failure in the intensive care units (ICUs) annually and up to 80% of them develop delirium during their ICU stay. Presence of delirium predisposes older adults to immediate in-hospital complications including a longer length of ICU and hospital stay, increased risk of in-patient mortality and elevated costs of care. In addition, ICU delirium is associated with long-term post-discharge complications such as development of cognitive impairment and dementia. Not only does the presence of delirium portend adverse patient-related outcomes; both delirium duration and delirium severity have also been identified as predictors of mortality and other adverse consequences. Research studies to date exploring pharmacological strategies to manage ICU delirium have not been successful in demonstrating efficacy. Additionally, receipt of sedative and opioid medications to treat other commonly co-occurring symptoms of pain and anxiety among the critically ill predisposes patients to further delirium propagation and persistence. Early implementation of a non- pharmacological intervention with the ability to manage pain and anxiety symptoms along with a reduction in harmful medications exposure holds great potential to reduce downstream delirium and its sequelae.

Music listening is one such intervention that holds promise for non-pharmacological management of delirium commonly experienced by mechanically ventilated patients. Music has been shown to break the harmful cycle of over-sedation in critically ill and can reduce anxiety and stress, factors that could predispose to ICU delirium. These findings are not surprising given that music results in a reduction in inflammatory cytokines, decreases cortisol production, and dampens central nervous system arousal through diminished norepinephrine release; pathways similar to those implicated in delirium. In a randomized clinical trial conducted by Dr. Chlan (MPI), a music intervention consisting of patient-directed, slow-tempo, relaxing music delivered during the latter portion of mechanical ventilator support was superior to usual ICU care in reducing anxiety and sedative exposure in critically ill patients. Even with such positive findings both at mechanistic and patient-outcome levels, music listening interventions are notably absent in routine ICU care. Further, there is a paucity of rigorous investigation of music’s impact on ICU delirium, a significant knowledge gap.

We now propose an adequately powered randomized clinical trial to test the efficacy of a seven-day music intervention in reducing delirium and improving brain health among critically ill, mechanically ventilated older adults through the following specific aims:

**Primary Specific Aim:**  Test the efficacy of music intervention in improving delirium/coma free days among mechanically ventilated patients as compared to attention control.

Hypothesis: Patients in the music intervention group will have higher number of delirium/coma free days as measured by the Confusion Assessment Method for the ICU (CAM-ICU) at seven days post randomization.

**Secondary Specific Aim 1:** Test the efficacy of music intervention in improving delirium severity, pain and anxiety among mechanically ventilated patients as compared to attention control.

Hypotheses: Patients in the music intervention group will have lower delirium severity, pain and anxiety scores as measured by the CAM- ICU-7, Critical Care Pain Observation Tool (CPOT) and Visual Analogue Scale-Anxiety (VAS-A) respectively at seven days post-randomization.

**Secondary Specific Aim 2:**  Test the efficacy of music intervention in improving the long-term neuro-psychological outcomes as compared to attention control.

Hypotheses: Patients in the music intervention group will have higher cognitive scores as assessed by the Indiana University Telephone Based Assessment of Neuropsychological Status (IU-TBANS), and lower depression and anxiety scores on the Patient Health Questionnaire (PHQ-9) and Generalized Anxiety Disorder (GAD-7) scales at 3 months post hospital discharge.

# Organization:

The data safety monitoring plan includes the appointment of an independent safety officer (SO) to perform data and safety monitoring activities. Dr. Noll Campbell, PharmD (Critical Care Delirium Researcher) will advise the PIs (Dr. Babar Khan, Dr. Linda Chlan) and the NIA program officer, regarding safety, study risks and benefits, scientific integrity, participant recruitment, and ethical conduct of the study.

1. **Qualifications and responsibilities of the Safety Officer (SO):**

The SO for this trial is a clinical researcher experienced in running intervention studies. The SO will review the reports sent by the study manager to determine whether there is any corrective action, trigger of an ad hoc review, or stopping rule violation that should be communicated to the study investigator, the Indiana University IRB, and the NIH. In addition, the SO may comment on whether the study investigators need to report any specific out of range data to the participant and/or her physician. SO is responsible for oversight of the safety of study participants and monitoring overall conduct of the study. The SO will review the protocol and consent, the safety parameters to be monitored, the frequency of committee monitoring reviews, and the study’s charter.

1. **Responsibilities of the DDM Study Team:**

The DDM research team will provide a package of introductory information to the SO, including the original protocol, consent, data safety monitoring plan charter, any amendments and research team contact information. The team will also provide an internal statistician to prepare reports for the SO. Furthermore, the team will notify the SO of any new significant safety information. The safety officer will serve as the first point of contact for adverse events (AEs) and serious adverse events (SAEs) for the study team. The safety officer will review all AEs and SAEs and determine if a safety monitoring meeting needs to convene.

1. **Conduct of Data Safety Monitoring Reviews:**

The DDM team will provide monthly safety reports to the safety officer. An annual report will be prepared and sent to the safety officer and the NIA

- - Following each annual safety report, the SO will make a recommendation to the DDM team to continue the trial or to terminate the trial early. This recommendation will be conveyed in writing within three working days of the report. If the recommendation is to stop the trial, then the area of concern (e.g. safety) should be first presented to the DDM team without breaking the blind. The Team has the final decision to stop the study and break the blind.
  - Data reviews by the SO will be distributed via email in password protected document files by the statistician or study coordinator. The SO is responsible for preventing access to these files by others at their institution. They will delete the files and shred any hard copy of the files to maintain confidentiality once their work on the project is completed.

The study coordinator and biostatistician will generate reports for PI and safety officer which contain:

a. Summary of adverse events and an explanation of how each event was handled

b. Summary of complaints and how each complaint was handled,

# Recommendations of the Safety Officer:

# At each annual review, after reviewing the data, the safety officer will recommend one of the following:

- - Continue the study unchanged;
  - Request further information from DDM team
  - Pause the study in order to address and further evaluate significant safety information

# Annual Review Documentation

All communications between the DDM Team and the safety officer will be documented and retained in the team’s DSMP file.

Copies of the annual report will be retained in a secured file for inspection by regulatory authorities (ie. IRB). They will be furnished upon request and for future reference by the safety officer. Recommendations will be signed by the safety officer and sent to the PI, who will then share them with the NIH and IRBs.

Enrollment in a study cannot begin until the safety officer’s recommendation for approval has been accepted by the NIA Program Office, and IRB approval has been obtained.

# Confidentiality

All members will treat as confidential all study related data and correspondence (the reports, meeting discussions and minutes).

# Expedited Safety Reporting Safety Reporting Plan

Clinical Outcomes (not considered Adverse Events): Critically ill patients and ICU survivors are at high risk for death or other adverse outcomes due to their underlying critical illness. Clinical outcomes, including death will be systematically tracked and will be included as part of the efficacy analyses for this study. For the purposes of reporting, death will not be recorded as adverse event unless the investigator believes the event may have been related to the study protocol (investigator’s discretion). This approach—considering death as outcomes rather than adverse events and systemically tracking expected safety outcomes for analysis rather than solely recording individual adverse events—is common in ICU trials because these outcomes/events occur commonly in the ICU/post-ICU population and this system mandates that data regarding death, and expected safety outcomes be tracked systematically for all patients and analyzed appropriately.

Clinical outcomes will be systemically tracked throughout the Study Period. Listed below are events that will be tracked as clinical outcomes and will not therefore be required to be reported as adverse events during this study (unless believed to be study intervention related and more severe or prolonged than expected):

- - Death. All deaths occurring within the Interventional Trial Phase will be reported on the CRF in the death summary section
  - Hospital or ICU readmissions
  - Depressive symptoms
  - Anxiety symptoms
  - Delirium-related outcomes including: trying to get out of bed, verbal abuse, falls, delayed procedures, pulling tubes, hospital-acquired pressure ulcers

# Adverse Events:

An adverse event (AE) will be any untoward medical occurrence for a patient enrolled in the trial that is not tracked as a clinical outcome. All AEs will be assessed as to whether related to the study protocol, serious and/or unexpected. These will include skin breakdown (around ears), cellulitis/skin infection (around ears) and allergic reaction (skin) to listening equipment.

**Related:** AEs that the investigators suspect are related to the study protocol.

**Serious:** AE, related to the study protocol, that result in any of the criteria below will be considered serious adverse event (SAE).

1. Death

2. A life-threatening episode requiring immediate intervention

3. Inpatient hospitalization

4. Persistence or significant incapacitation or substantial disruption of the ability to conduct normal life functions

5. An episode that requires intervention to prevent the above and/or permanent impairment or damage

**Unexpected:** AEs, including SAEs, related to the study protocol which are more severe or prolonged than expected will be considered unexpected.

# Measurement and reporting of adverse events:

Adverse event rates associated with music intervention are low. Therefore, adverse event rates are expected to vary little between the treatment and control groups. We will present blinded adverse event data to the statistician and PI throughout the trial. We plan to present unblinded adverse events data when requested by the safety officer. If there is evidence of elevated adverse events, the safety officer will consult with the statistician and PI. The study staff will use an adverse event form to report adverse events caused by the intervention.

The PI will be notified within 24 hours of identifying the occurrence of any possible adverse events. Serious and Unexpected adverse events will be reported within 5 business days to IU IRB, Safety Officer, and NIH. Non-serious adverse events will be reported at time of continuing review to IU IRB.

The information collected will include the following:

- - Description of the AE.
  - Time and Date of onset and eventual date of resolution or death, if available.
  - Frequency: intermittent, single occurrence, or continuous.
  - Severity of the event.
  - Action taken, including use of any concomitant treatment or medication.
  - Outcome.
  - Relationship to study treatment regimen or other causality.

**Stopping Rules:** It is unlikely that the study would be stopped early due to important favorable differences in the intervention group compared to control group because of the short-term nature of the intervention. However, the study could be stopped early due to adverse events. The NIA will make the final decision on whether or not to accept the Safety Officer’s recommendation about discontinuation of any component of the study.

**Limits of Assumptions:** It is possible that baseline differences between the groups, excessive study dropouts and/or missing data by the interim measurement time point (midway point to targeted enrollment) will limit the value of data analysis. Baseline differences will be evaluated after the first measurement time point and effects on the power to detect differences in the primary outcome will be evaluated and communicated to the PI, SO, and NIA. It is exceedingly unlikely that there will be baseline differences between groups of any magnitude to threaten the validity of the study.

1. **PROTECTION OF HUMAN SUBJECTS**
2. **Risks to Human Subjects:**
   1. Human Subjects Involvement, Characteristics, and Design**:** 80% of older adults develop delirium during their ICU stay. Presence of delirium predisposes older adults to immediate in-hospital complications including a longer length of ICU and hospital stay, increased risk of in-patient mortality and elevated costs of care. In addition, ICU delirium is associated with long-term post-discharge complications such as development of cognitive impairment and dementia. Our team is proposing to conduct a randomized clinical trial called “Decreasing Delirium through Music (DDM) in Critically Ill Older Adults” to evaluate the efficacy of a seven-day slow-tempo music intervention on the primary outcome of delirium/coma free days among mechanically- ventilated older adults admitted to the ICU.

The target population will be English-speaking; mechanically ventilated adults aged 50 and older who have been admitted to the intensive care unit in either Eskenazi Health or Methodist Hospital in Indianapolis. The intervention comprises slow-tempo relaxing music versus a silence track attention control delivered twice daily for seven days. The primary outcomes will be delirium/coma free days. The enrolled population will also include women and minorities who meet the inclusion criteria. Subjects will be excluded as follows: [1] History of dementing illnesses and other neurodegenerative diseases such as Alzheimer’s disease or vascular dementia; [2] Psychiatric illness which is not well controlled [3] Alcohol withdrawal symptoms/concern for withdrawal; [4] Suspected or confirmed drug intoxication/overdose, [5] Traumatic brain injury, ischemic or hemorrhagic cerebrovascular accident, or undergoing neurosurgery; [6] Uncorrected hearing or vision impairment including legal blindness; [7] Incarcerated at the time of study enrollment, [8] Enrolled in another clinical trial which does not permit co-enrollment

After obtaining approval from the local institutional Review Board (IRB), subjects or their legally authorized representative will be approached for participation into the study. After enrollment the subjects will be randomized into two groups, slow-tempo music intervention and silence track attention control. The interventionist research manager will initiate the study intervention. The research staff will perform blinded outcomes assessment including delirium/coma free days, delirium severity, pain, and anxiety during the ICU stay. After discharge, at 3 months, research assistants will perform cognition assessment, and will collect depression and anxiety measures over the phone.

- 1. Sources of Materials**:** During the hospital stay, research assistants will review the medical records and conduct delirium, coma, pain, and anxiety assessments. The blinded research assistants will also be collecting information on physiologic status (blood pressure, height, weight, heart rate, respiratory rate). The assessors will also conduct a medical record review to assess subject’s chronic conditions (Charlson comorbidity index) and severity of medical illness (APACHE II index, SOFA).

All data gathering is done initially with subjects or their legally authorized representatives in the ICU and afterwards at the 3 months post hospital discharge follow-up. Data will be linked to participants through the use of a unique identifying number. Only persons on the research team will have access to the data. All data are collected for research purposes only. Case Report Forms (CRFs) will be stored in locked filing cabinets at Regenstrief Institute and all data will be entered into electronic case report forms (eCRFs) in a secured password-protected database. All study data will be entered via a password-protected, study specific REDCap (Research Electronic Data Capture) database website. REDCap was developed specifically around HIPAA- Security guidelines and has been disseminated for use locally at other institutions and currently supports > 140 academic/non-profit consortium partners and 11,000 research end-users (www.project-redcap.org).

- 1. Potential Risks**:**

1. *Fatigue, anxiety, stress, or embarrassment from the assessments.* Emotional distress may result from answering health and behavior questions. Testing may also create anxiety, stress, or embarrassment at perceived performance. Participants may also become fatigued during the testing.
2. *Exposure of confidential information.* There is the potential for loss of privacy or confidentiality due to the data collection efforts of this study.
3. *Discomfort with music delivery.* There is potential for discomfort, infection, and irritation with use of the headphones. Another potential risk is that a subject could have an adverse emotional response to a certain piece of music. The potential risk for an adverse emotional response to music is thought to be low in regard to the music planned for our study playlist. Our music therapist consultant has extensive professional experience with ICU patients receiving mechanical ventilation.
4. *Depression and suicidal ideation.* The duration of the study intervention is limited to the ICU; therefore, adverse events related to the intervention occurring after the date of discharge are unlikely. Because research staff will continue to collect data from participants for 3 months after discharge, and because psychological complications have been described in ICU survivors, study staff will recognize worrisome conditions such as suicidal ideation or other emergent concerns of clinical conditions (chest pain, etc.) during any study interaction. Study personnel will follow a scripted protocol to notify the appropriate medical personnel and the PI so that appropriate notification and clinical follow-up is ensured. The PI or delegated study personnel will notify the appropriate provider or emergency personnel depending on the severity of the presentation. Even in depression trials conducted by Regenstrief investigators with clinically depressed patients, this is only an occasional event, and in the present study we expect it to be rare. Nonetheless, we will train study personnel in the recognition and communication of such events to ensure prompt response to emergent reports if they occur.
5. *Discomfort with blood draws.* There is potential for discomfort or localized irritation due to blood draws.
6. **Adequacy of Protection Against Risks:**
   1. Recruitment and Informed Consent: Eligible subjects will be identified through the intensive care units census to which they are admitted. A waiver of consent documentation will be obtained for recruitment purposes only. Study personnel will consent the patient or their legally authorized representative (if the patient is unable to consent for themselves).
   2. Protections Against Risk**:**
7. *Fatigue, anxiety, stress, or embarrassment from the training or testing sessions.* All questions planned for this study are part of validated standardized instruments, and we are not asking any questions that do not directly relate to the study purpose. Research staff will be trained in their proper use and in the importance of privacy and sensitivity to the participant’s time. They will be trained to be alert and sensitive to signs of fatigue and other symptoms and to take appropriate actions when they are present.
8. *Exposure of confidential information.* Indiana University requires certification of training in protection of human subjects in research. The investigators, interventionist, assessors, and all key personnel have or will have successfully completed training and certification in these courses. All research involving the use of these data must be reviewed and approved by the IRB. We will assure the privacy of subjects and confidentiality of study data by assigning unique identifiers to track participants’ data (rather than using names or hospital or social security numbers) and keep all records under lock with access only by study personnel. These procedures have been dutifully adhered to in prior studies. The final data files for this study will be merged, maintained, and analyzed on servers managed by the Division of Biostatistics, Department of Medicine, Indiana University School of Medicine. We will also utilize these rigorous protocols to protect confidentiality of biomarker analyses which include genetics and epigenetics associations. Our group has extensive experience in the handling and security of PHI. None of the individual participant data will be identifiable in published reports or manuscripts and the analyzable datasets will not contain the participant’s unique identifier.
9. *Discomfort with music delivery.* Patients will be monitored for adverse events on a daily basis. Adverse events will be reported to the Institutional Review Board (IRB), data safety officer, and PIs immediately. In our prior work, we have not experienced any adverse events related to the study protocols. All infection control guidelines will be followed based on each hospital’s policy. Patient risks will be minimized through the use of quality electronic devices, their regular cleaning and maintenance. If patients endorse discomfort from headphones, they will be switched to another set. In case of an adverse emotional response to music, subjects will be informed that they are free to withdraw from the study at any time they so desire without affecting their care. In previous work by the PI in which subjects listened to music from a discrete collection, no adverse emotional responses to music has occurred, thus we believe our plan here will be equally effective.
10. *Depression and suicidal ideation*: During follow-up assessments it is possible that participants endorse suicidal ideation on the PHQ-9. We have handled them extensively in our prior m-CCRP and IMPROVE trials and have developed a suicide response protocol. A positive PHQ-9 will be followed by the Columbia Suicide Severity Rating Scale (C-SSRS) questions. If a participant answers yes to any C-SSRS question, the PI (Dr. Khan) will be notified immediately who will contact the family member to arrange transfer to the ER. If the participant does not answer yes to any C-SSRS question, the RA will still notify Dr. Khan who will contact the PCP of the patient and will arrange for an urgent follow-up.

(5) *Risks related to blood sample collection* will be minimized by use of clinical nurses for sample collection, use of existing intravascular devices for drawing blood whenever possible, and transport of the samples using lab best practices, and labelling the samples with deidentified labels.

# 
