# Supplementary material for: Decreasing delirium through music listening (DDM) in critically ill, mechanically ventilated older adults in the intensive care unit: a two-arm, parallel-group, randomized clinical trial
Source: Trials. 2022 Jul 19;23:576. doi: 10.1186/s13063-022-06448-w (PMC9295531; doi:10.1186/s13063-022-06448-w)
Supplement: Supplementary file 2 — Additional file 2. [file 13063_2022_6448_MOESM2_ESM.docx]

# INDIANA UNIVERSITY INFORMED CONSENT STATEMENT FOR

Decreasing Delirium Through Music in Critically ill Older Adults (DDM)

You are invited to participate in a research study of music to reduce confusion (also called delirium) in the intensive care unit.

You are being asked to participate as you are admitted to the intensive care unit. Scientists do research to answer important questions which might help change or improve the way we do things in the future. This consent form will give you information about the study to help you decide whether you want to participate.

Please read this form, and ask any questions you have, before agreeing to be in the study.

**STUDY PURPOSE**

The purpose of this study is to measure the effects of music on delirium (confusion). We will also measure the effects of music on pain, anxiety, mood symptoms, and your ability to perform memory and thinking tasks.

**NUMBER OF PEOPLE TAKING PART IN THE STUDY**

If you agree to participate, you will be one of about 160 people taking part in the project.

**PROCEDURES FOR THE STUDY**

If you agree to be in the project, the following will occur:

- You will be asked to provide information on your favorite types of music genres (for example, pop, country, hip/hop or soul music etc.).
- You will be randomly assigned to one of two groups:
  - You may hear music from a library of music provided to you
  - You will receive a “silence track” that blocks out noise from your hospital room.
- During your stay in the ICU, you will receive either music or a silence track for one hour sessions twice a day for up to 7 days. You will hear this audio program through headphones and a music player (both of which we will provide for the duration of the study).
- We will monitor you for symptoms or signs of confusion, pain, anxiety during the course of your ICU/hospital stay with assessments that can occur up to 4 times a day. You will be assessed with questionnaires asking you simple questions and exercises involving thinking and memory.
- A blood draw from a vein in your arm or hand will be completed 3 times, totaling about 2 tablespoons :
  - prior to the first music or silence track session
  - on day 3 of receiving the music or silence track sessions
  - after the last music or silence track session
- We will record data from your medical record/chart. This data includes but is not limited to vitals, lab values, major events, x-ray and other radiology results, medications and doses.
- We will call you about 3 months after your discharge from the hospital to complete a questionnaire that measures your memory and thinking ability (brain function), mood and related symptoms.
- It is not uncommon for patients recovering from the ICU to develop depression. If your mood symptoms indicate a risk of self-harm or suicide, we may have to report this to your doctor or emergency medical services.

Participation or non-participation in this study will not change your treatment by the medical or surgical teams, including which medications you are administered.

**RISKS OF TAKING PART IN THE STUDY**

The risks of participating in this project include:

1. You may feel tired, anxious, stressed or embarrassed during bedside assessments which will occur twice a day. If this occurs, let the research staff know and we will do our best to accommodate your needs.
2. You could experience discomfort or skin irritation with the headphones. You could experience discomfort with the songs if they are not to your liking or if they trigger an emotion. If this occurs, we can change the headphones and/or the music/songs for you.
3. While we take great effort to protect your private information, loss of confidentiality (your private health details becoming known to people who are not part of your healthcare or research team) is a risk.
4. You may have pain, bruising or vary rarely get an infection from the blood draw. To minimize these problems, blood will be drawn by trained staff using sterile procedures.

**BENEFITS OF TAKING PART IN THE STUDY**

While there are no guarantees that you will benefit from your participation, based on prior research studies, we feel that participation in this project will provide you a new experience and the music may decrease your level of anxiety in the intensive care unit.

**ALTERNATIVES TO TAKING PART IN THE STUDY**

You may choose not to participate.

You will not miss out on any treatments or benefits by not joining this study or dropping out once it has started.

The medical or surgical teams will not treat you differently regardless of your participation.

**WILL I RECEIVE MY RESULTS?**

At the end of our research study, and once all analysis is performed, we can provide you with a summary of our findings; however, these may not be your individual test results.

**CONFIDENTIALITY**

Efforts will be made to keep your personal information confidential. We cannot guarantee absolute confidentiality. Your personal information may be disclosed if required by law. Your identity will be held in confidence in any published reports and databases.

Organizations that may inspect and/or copy your research records for quality assurance and data analysis include groups such as the study investigator and his/her research associates, the Indiana University Institutional Review Board or its designees, and (as allowed by law) state or federal agencies who may need to access your medical and research records.

For the protection of your privacy, this research is covered by a Certificate of Confidentiality from the National Institutes of Health. The researchers may not disclose or use any information, documents, or specimens that could identify you in any civil, criminal, administrative, legislative, or other legal proceeding, unless you consent to it. Information, documents, or specimens protected by this Certificate may be disclosed to someone who is not connected with the research:

(1) if there is a federal, state, or local law that requires disclosure (such as to report child abuse or communicable diseases);

(2) if you consent to the disclosure, including for your medical treatment;

(3) if it is used for other scientific research in a way that is allowed by the federal regulations that protect research subjects;

(4) for the purpose of auditing or program evaluation by the government or funding agency;

A Certificate of Confidentiality does not prevent you from voluntarily releasing information about yourself. If you want your research information released to an insurer, medical care provider, or any other person not connected with the research, you must provide consent to allow the researchers to release it.

A description of this clinical trial will be available on [ClinicalTrials.gov](http://clinicaltrials.gov/), as required by U.S. law. This website will not include information that can identify you. At most, the website will include a summary of the results. You can search this website at any time.

**USE OF YOUR DE-IDENTIFIED INFORMATION IN FUTURE RESEARCH**

Information collected from you for this study may be used for future research studies or shared with other researchers for future research. If this happens, information which could identify you will be removed before any information is shared. Since identifying information will be removed, we will not ask for your additional consent.

**GENETICS INFORMATION**

**This research follows the Genetic Information Nondiscrimination Act, a federal law that generally makes it illegal for health insurance companies, group health plans and most employers to request the genetic information we get from this research and to discriminate against you based on your genetic information.**

**We may us the specimens collected as a part of this study for whole genome sequencing, which involves mapping all of your DNA.**

**COSTS**

There is no cost to you or your insurance company for any procedures in this project.

**PAYMENT**

There is no payment associated with participation in this study.

**COMPENSATION FOR INJURY**

In the event of physical injury resulting from your participation in this research, necessary medical treatment will be provided to you and billed as part of your medical expenses. Costs not covered by your health care insurer will be your responsibility. Also, it is your responsibility to determine the extent of your health care coverage. There is no program in place for other monetary compensation for such injuries.

However, you are not giving up any legal rights or benefits to which you are otherwise entitled.

**CONTACTS FOR QUESTIONS OR PROBLEMS**

For questions about the study or a research-related injury, contact the researcher Dr. Babar Khan at 317-274-9427. If you cannot reach the researcher during regular business hours (8:00 AM-5:00 PM), please call the IU Human Subjects Office at 317-278-3458 or 800-696-2949.

For questions about your rights as a research participant or to discuss problems, complaints or concerns about a research study, or to obtain information, or offer input, contact the IU Human Subjects Office at 317-278-3458 or 800-696-2949.

**VOLUNTARY NATURE OF STUDY**

Taking part in this project is voluntary. You may choose not to take part or may leave the project at any time. Leaving the project will not result in any penalty to you or loss of benefits. If you decide not to participate in this project, it will not affect your current or future relations with Eskenazi Health or Indiana University Health.

The project staff may stop your participation in the listening sessions if you become extremely anxious or uncomfortable. This will be done to protect your health as well as the training experience for the other participants.

# The project is reviewed at regular intervals by a panel of outside scientists. They review project records for signs of troublesome side effects associated with participation such as study-caused injuries. If problems are detected, the investigator and the review board will work to determine if the project should be stopped.

**CAN I WITHDRAW FROM THE STUDY?**

If you decide to participate in this study, you can change your mind and decide to leave the study at any time in the future. The study team will help you withdraw from the study safely. If you decide to withdraw, you should let a study team member know.

**SUBJECT’S CONSENT**

In consideration of all of the above, I give my consent to participate in this research project.

I will be given a copy of this informed consent document to keep for my records. I agree to take part in this project.

**Subject’s Printed Name: _______________________**

**Subject’s Signature**: **Date**:

(must be dated by the subject)

**LAR’s Printed Name, as applicable: ____ ____________________**

**LAR’s Signature, as applicable**: **Date**:

(must be dated by the LAR)

**Printed Name of Person Obtaining Consent: _______________________**

**Signature of Person Obtaining Consent**: **Date**:
